# Electron Beam Irradiation on the Production of a Si- and Zr-Based Hybrid Material: A Study by FTIR and WDXRF

**DOI:** 10.3390/ma16020489

**Published:** 2023-01-04

**Authors:** Alexandra P. Rodrigues, Pedro M. P. Santos, João Pedro Veiga, Maria Helena Casimiro, Luís M. Ferreira

**Affiliations:** 1 Centro de Ciências e Tecnologias Nucleares (C2TN), Instituto Superior Técnico, Universidade de Lisboa, Estrada Nacional 10, km 139,7, Bobadela, 2695-066 Loures, Portugal; 2 Departamento de Conservação e Restauro (DCR), Faculdade de Ciências e Tecnologia, Universidade NOVA de Lisboa, Campus de Caparica, 2829-516 Caparica, Portugal; 3 CENIMAT/i3N, Centro de Investigação de Materiais, Faculdade de Ciências e Tecnologia, Universidade NOVA de Lisboa, Campus de Caparica, 2829-516 Caparica, Portugal; 4 Departamento de Engenharia e Ciências Nucleares (DECN), Instituto Superior Técnico, Universidade de Lisboa, Estrada Nacional 10, km 139,7, Bobadela, 2695-066 Loures, Portugal

**Keywords:** hybrid materials, PDMS, TEOS, zirconium, radiation processing, electron beam

## Abstract

Sol-gel production of hybrid materials has, to some extent, revolutionised materials’ engineering and the way science and technology perceive the creation of new materials. Despite that, the method presents some limitations that are circumvented by radiation processing. Electron beam irradiation was used to promote synthesis of hybrid structures while using silanol-terminated PDMS, TEOS and TPOZ as precursors. Evaluation of the method’s performance was executed by gel fraction determination, WDXRF and FTIR-ATR. Results showed that, although there is some pre-irradiation reactivity between precursors, radiolysis induces scission on multiple sites of precursor’s structures, which induces hybrid network formation to a greater extent. Characterisation allowed determining electron beam irradiation to be effective in the creation of Si–O–Zr bonds, resulting in the production of a Class II hybrid material.

## 1. Introduction

Hybrid materials (HMs) are an example of technology’s contribution to the evolution of societies and civilisations. HMs can be found in nature (bone, nacre and wood) but humans have prepared artificial ones since time immemorial. Some examples are the pre-history’s Lascaux’s hybrid paints, antiquity’s Maya blue dye, the Chinese rice–lime mortars and the modern age’s Prussian Blue pigment. Researchers have been inspired by the widespread existence of these materials, and so research and development in this field started a few decades ago. The understanding of the chemistry involved and the control over the preparing processes have enormously extended the variety and versatility of designed HMs for a wide range of applications; however, special attention has been devoted to silicon-based HMs since the 17th century, when the first silicates’ gelation experiments took place [1,2,3].

Since then, the majority of novel HMs has been produced by sol-gel methods. In 1985, in the United States of America, silicon-based precursors—polydimethylsiloxane (PDMS) and tetraethylorthosilicate (TEOS)—were used for the first time to form a class II hybrid material by sol-gel methods. This designation is attributed to HMs whose structures are composed exclusively or partially by covalent, by iono-covalent, or by Lewis acid-base bonds linking the organic and inorganic moieties. Further materials were developed with the goal of incorporating transition metals oxides in PDMS–TEOS hybrid matrices [2,3]. These offer many options, from production to applications, covering a wide range, such as the following: the production of biomedical implants [4], coatings to prevent corrosion of chrome-coated steel [5], the production of pollutants sensors [6], or the consolidation of ceramic [7] and stone [8] in cultural heritage.

Yet, the sol-gel method presents several conditioning factors, such as the need of the addition of further reactants acting as catalysts. Often, these substances can be harmful for the operators; moreover, they can also be responsible for unwanted experimental side effects that interfere with the functional viability of produced materials. Most particularly regarding the method of nanoparticles in situ formation in a polymeric matrix, sol-gel produces materials with characteristic porosity caused by the evaporation of solvents and catalysts which sometimes is not desirable [9]. In addition, the presence of water may prevent the integration of transition metals in polymer matrices by causing their precursors’ precipitation, as is the case of zirconium tetrapropoxide (TPOZ) in the PDMS–TEOS system [10,11].

Circumventing these limitations, alternative methods to induce the hybridisation—i.e., the synthesis of hybrid structures from chosen precursors [12]—have been developed in the recent past.

Two-photon polymerisation has been used as a method of fabricating three-dimensional structures with resolutions below 100 nm based on inorganic–organic HMs with multiple applications. This method is fast and flexible when contextualised with the scale of its action and provides minimal shrinkage during and after polymerisation. The low scale and volumes of processed materials are significant disadvantages though [13,14,15].

Former work by C2TN (Formerly identified as Instituto Tecnológico e Nuclear and integrated in Instituto Superior Técnico from Universidade de Lisboa, since 2012.) has instead focused on the production of HMs prepared by gamma irradiation (γ-HM) [10,16,17,18,19,20]. Radiation-induced polymerisation for hydrogels and other hybrids is a recent trend in this field. In this method, hydrolysis and subsequent condensation typical of sol-gel are replaced by radiolysis induced by gamma photons and subsequent recombination between generated fragments. The corresponding line of research has created materials with very interesting optical, mechanical and structural properties, namely transparency, cohesion, hydrophobicity or high thermal stability, spanning from glass-like materials to more elastomeric ones. The proven advantages of this method include the following:

- catalysts not needed to promote cross-linking;
- no solvents or water required;
- residues after hybrid network formation are reduced;
- higher cross-linking degree due to presence of more activated sites on precursors structures generated by radiolysis.

However, the preparation of HMs requires considerably high doses. This is often a drawback when γ-radiation is used due to the very long irradiation time needed and low radioresistance of samples’ containers to highly accumulated doses.

Directing this work for applications in the cultural heritage field, it is possible to determine that there are recurrent deterioration forms affecting different types of historical materials that require multiple types of interventions. Loss of cohesion of porous materials such as ceramics, stone and mortars features as one the most problematic, along with loss of adhesion related to layered materials. Typically, this is the result of interaction with deteriorating agents such as water, pollutants, biological agents or exposure to mechanical stress [21]. Therefore, conservation-restoration interventions aim many times at consolidating and fixating materials and structures, while at the same time, it is necessary to resort to biocides or biocolonisation preventers and water repellents in order to minimise recurrence of deterioration. It is thus understandable that this implies procedures involving several techniques and materials. By resorting to a multifunctional material that can address several of these issues simultaneously, conservation-restoration interventions can become less time-consuming and requiring less human resources, therefore becoming less costly.

Centring the discussion on the PDMS–TEOS system, there are specific properties of the precursors that are appropriate in a material developed for this means. PDMS shows suitable flexibility, with elastomeric properties, transparency and lack of colour. It is also hydrophobic and presents high thermal stability and low susceptibility to degradation by agents such as ultraviolet light, oxygen or ozone [22]. Nevertheless, it presents poor structural properties and its biocompatibility [23] may, ironically, be incompatible with the objective of being used in the formulation of a material that should prevent biodeterioration. TEOS, on the other hand, has been widely used as a consolidant of historical materials and more occasionally on the formulation of protective coatings also employed on cultural heritage [7,24,25,26,27]. This alkoxide (ALK) enables the network formation of PDMS-based materials and provides it structural properties besides enhancing the consolidation effect.

Building on this, the authors aimed at developing the same type of multifunctional material, resorting to electron beam irradiation. The advantages of this process include a more expedite control of dose rates, more safety for the operators and a very significant time economy, since much higher dose rates are easily achieved than with gamma irradiation, which decreases total processing time. The use of this method for the production of PDMS–TEOS hybrid materials and taking advantage of shorter irradiation times is possible mainly because there is little to no dependence of the PDMS–TEOS system on dose rate, as shown by a previous study [17].

Given the goal of conferring biocide or biocolonisation prevention properties to the final product and the aforementioned biocompatibility of PDMS, it was necessary to introduce a third precursor in the formulation. Based on the antimicrobial properties of zirconium [28,29,30] and the group’s previous experience with Zr in PDMS–TEOS prepared by γ-irradiation, TPOZ was the choice of Zr precursor to be used.

## 2. Materials and Methods

### 2.1. Materials

**Figure 1:**
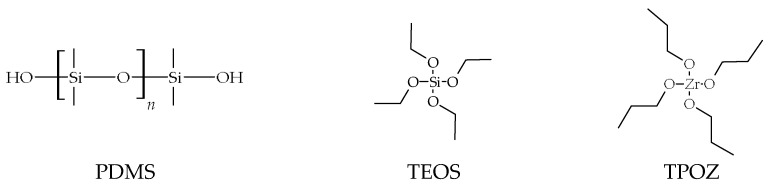
Precursors’ structures.

The precursors were used as received from the suppliers (Figure 1):

- Silanol-terminated PDMS, with 0.8 wt% in OH, with molar mass 43,500 g·mol^−1^ and 3500 cSt viscosity (S33) (ABCR GmbH, Karlsruhe, Germany)
- TEOS (Si(OCH_2_CH_3_)_4_) (Aldrich, St. Louis, MO, USA)
- TPOZ (Zr(O(CH_2_)_2_CH_3_)_4_), 70 wt% solution in 1-propanol (Aldrich)

### 2.2. Choice of Formulation

The choice of the best formulation was based on compositions previously produced using gamma irradiation and already characterised from the point of view of morphology, composition, structure and thermal behaviour [16,31].

Given the objective of conferring biocidal properties on the material produced, a preliminary assay was carried out to identify the formulation with the best performance in this scope, evaluating the intrinsic bioactivity of these materials on selected microbiological contaminants. Best results were achieved by compositions with [TPOZ] = 20%. Formulations with mass ratios PDMS/ALK < 1 result in powdery samples, without cohesion, due to the absence of connections between the inorganic regions and the polymeric network [10]. For this reason, the chosen formulation was PDMS:TEOS:TPOZ with 67:13:20 m% (Precursors ratio is expressed in mass percentage (m%) and not weight percentage (wt%), since all the precursors quantities necessary for hybrids preparation were measured using an analytical electronic balance with internal calibration, which gives direct reading of samples masses), respectively—mass ratio PDMS/ALK > 2—since this resulted in monolithic, flexible, homogeneous, transparent and colourless materials [10]. Irradiations were also performed on PDMS samples to determine a dose threshold that could guarantee gelation in irradiated mixture samples.

A sample which was previously prepared by γ-irradiation was also used for comparison in this study, specifically for the gel point determination of electron beam-irradiated PDMS. The sample was irradiated in the ^60^Co irradiator facility of Instituto Superior Técnico. The attained dose was 700 kGy at a dose rate of 30 kGy·h^−1^.

### 2.3. Samples Preparation and Conditioning

Precursors were mixed by alternating mechanical stirring with a vortex mixer and ultrasound, keeping the vessel in a water bath at normal temperature.

Aliquots of ~6 mL of the mixture making up to 5 mm in height were poured into colourless transparent polystyrene (PS) boxes with square bases with ~3 cm sides. These were then flooded with gaseous N_2_, closed and sealed inside low-density polyethylene (LDPE) bags also flooded with N_2_, making sure the bag would not be inflated enough to affect irradiation geometry.

**Figure A1:**
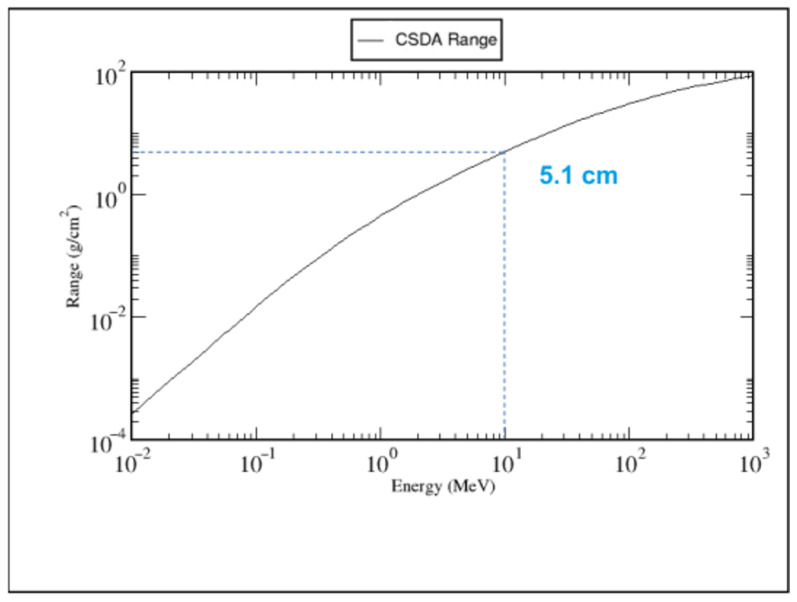
Simulation results depicting the range of electron beams with ~90 eV mean excitation energy in PDMS:TEOS:TPOZ with 67:13:20 m% proportions.

To minimise pre-irradiation reactions between the precursors, the already sealed samples were maintained at a temperature < 10 °C until placement in targeted position. Please address Appendix A and Appendix B for further details about the preparation and irradiation procedures and, in Figure A1, about beam range through Mix simulated with ESTAR [32]. 

Samples’ names are attributed according to their composition and status as described in Table 1:

Example 1:

γ-PDMS700

Gamma photons irradiated polydimethylsiloxane with accumulated dose of 700 kGy.

Example 2:

e-Mix215

Electron beam irradiated mixture of PDMS:TEOS:TPOZ with a concentration of 67:13:20 m% as of preparation, and accumulated dose of 215 kGy.

Example 3:

air-Mix

In-air cured, non-irradiated mixture of PDMS:TEOS:TPOZ with a concentration of 67:13:20 m% as of preparation.

### 2.4. Irradiation Parameters

Irradiations were performed in a linear electron accelerator (LINAC, adapted from GE Saturne 41, EuroMeV, Paris, France) located at the Ionizing Radiation Facility (IRIS) from C2TN/IST-UL. The system was set to a 10 MeV electron beam whose peak current was 50 mA. The pulse width and repetition frequency were 4 μs and 25 Hz, respectively. Average beam power was 50 W. Medium dose rate at target, Z=16 cm, was approximately 7.3 kGy·min^−1^ varying between 6.49 kGy·min^−1^ and 8.34 kGy·min^−1^.

The absorbed doses were estimated using calibrated radiochromic films FWT-60 Far West Technology, Goleta, CA, USA. The routine dosimeters were placed between the LDPE bag and the PS plate sample holder.

Samples were irradiated for periods up to 55 min, in partial exposure times between 2 min and 12 min 25 s of continuous irradiation, to minimise temperature increase and effect. Temperatures after partial irradiations reached values between 29 °C and 42 °C. The samples were cooled between partial irradiation exposures by subjecting them to $T\;\sim -10\;{}^{{}^{\circ}}C$, until they reached $T<10\;{}^{{}^{\circ}}C$.

Ten aliquots were irradiated, reaching different absorbed doses to assess the evolution of the final product. Attained doses were 68, 113 and 213 kGy for e-PDMS and 70, 99, 132, 154, 178, 215, 248, 320, 370 and 388 kGy for e-Mix. The uncertainty associated to the absorbed doses was 4%.

### 2.5. e-Mix Characterisation

#### 2.5.1. Gel mass Fraction Determination

Gel mass fraction was determined by calculating the ratio between mass of the sample after processing and mass of the sample before processing [33,34], according to

(1) $$Gel\;mass\;fraction\;\%=\frac{mass\;after\;processing_{}}{mass\;before\;processing_{}}\times 100.$$

Samples processing was composed of the following steps:

1. Extraction of unreacted materials and precursors’ fragments by immersion in tetrahydrofuran for 72 h;
2. Evaporation of extraction solvent and extracted substances by drying in air for 6 days;
3. Drying at 80 °C for 12 h in a laboratory oven, to guarantee non-matrix materials’ evaporation.

#### 2.5.2. Wavelength Dispersive X-ray Fluorescence

Wavelength dispersive X-ray fluorescence (WDXRF) analyses were executed in CENIMAT/FCT-NOVA with a PANalytical XRD-WDS 4 kW AXIOS sequential spectrometer equipped with a rhodium tube (Almelo, The Netherlands).

#### 2.5.3. Fourier Transform Infrared Spectroscopy

Chemical bonds in e-HMs were studied by Fourier transformed infrared spectroscopy (FTIR) in attenuated total reflectance mode (ATR). Acquisition was performed in the range 4000–400 cm^−1^, with resolution of 4 cm^−1^, for 64 scans. These analyses were performed with a Nicolet iS50 spectrometer by Thermo Scientific (Waltham, MA, USA).

## 3. Results

### 3.1. Gel Fraction and Gel Point Determination

Irradiated PDMS samples were flowing after 68 kGy of accumulated dose, although very viscous and apparently non-flowing at D=113 kGy. Gel mass fractions for these doses corresponded to 89.5% and 99.5%, respectively, indicating a significant increase in the number of created bonds in the matrix between the two dose values. The third dose, 213 kGy, corresponds to 99.4% gel mass fraction, showing no significant difference in the influence of accumulated dose on gel mass fraction regarding the previous point and, therefore, in crosslinking promotion. These factors corroborate the estimation of gel point of PDMS to be in the 68–113 kGy range of accumulated dose. Given this, this range was considered to be the threshold dose, above which it would be possible to achieve gel point in the 67:13:20 m% mixture of PDMS:TEOS:TPOZ.

**Figure 2:**
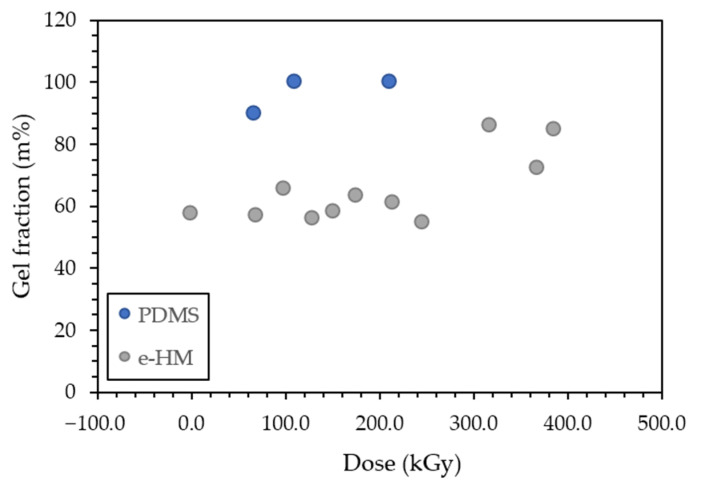
PDMS and e-Mix determined gel fraction (m%) as a function of absorbed Dose (kGy).

It is evident in Table 2 and Figure 2 that none of the e-Mix samples presents a gel mass fraction close to 100%, in opposition to what happens with e-PDMS, which means that there was no complete gelation with the used doses. This also implies the lingering presence of fragments of the precursors in all irradiated materials, regardless of accumulated dose. There is, however, a significant increase in these values, from 53.8% to 85.5%, for materials irradiated with accumulated doses of 248 kGy and 320 kGy. This could point to this range as comprising gel point for e-Mix. Nevertheless, materials with accumulated doses above 388 kGy, should be studied in the future to clarify this point.

### 3.2. WDXRF Spectroscopy

Si/Zr ratios in atomic fraction (χ_at_) calculated from WDXRF data represented in Table 3 show that all samples, with exception of e-Mix215, exhibit a lower content in Si than the theoretical value calculated for the as-prepared mixture. This decrease, according to Gomes et al. [17], is in all probability the reflection of evaporation of TEOS before irradiation, causing Zr content in each sample to apparently increase. Si content decrease occurs also in air-Mix, strengthening this hypothesis.

It is also visible that e-Mix irradiated with 70, 132, 154, 178, 320, 370 and 388 kGy present a higher decrease in Si content than air-Mix. This suggests these samples may have further lost Si after irradiation, meaning the latter may have recombined with volatile fragments produced by radiolysis and evaporated due to not being incorporated in the post-irradiation matrix. Another conclusion that can be drawn is that the higher stoichiometry favouring Zr content is achieved with 320 kGy, originating a network of approximately 1 atom of Zr to 4.12 atoms of Si proportion.

To understand whether Zr was incorporated in the network or not, WDXRF was again performed on samples after processing for unreacted materials and fragments’ extraction.

**Figure 3:**
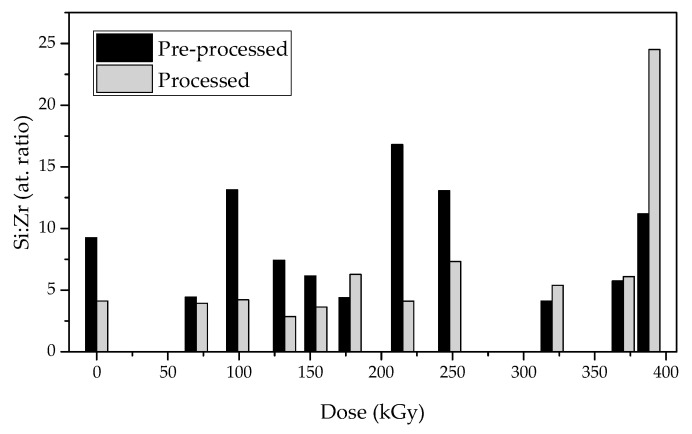
Si:Zr atomic ratio before and after processing for unreacted materials’ and fragments’ extraction.

The results in Table 4 and Figure 3 show that when comparing the samples before processing_,_ there is an increase in Zr content in air-Mix and irradiated samples with up to 248 kGy, except e-Mix178, after processing. Like the exception, samples irradiated with doses 320–388 kGy present lower Zr contents. Higher Zr contents after extraction processing infer that the unreacted materials and fragments that are extracted after irradiation, and therefore never formed covalent bonds with the produced network, are richer in Si than in Zr. The remaining material is, opposingly, richer in Zr and the higher stoichiometry-favouring Zr content is achieved with 132 kGy, originating a network of approximately 1 atom of Zr to 4.12 atoms of Si proportion. The seemingly trend inverts after 248 kGy. For the doses above this value, and for 178 kGy, the Zr content decreases after processing, implying the extraction removes fragments that are richer in Zr than in Si content.

If sample e-Mix178 is considered an outlier, these values suggest that extracted Si-rich fragments are the result of PDMS and TEOS pre-irradiation alterations and that doses ≥ 320 kGy cause the degradation of the produced matrix by breaking it and producing more Zr-rich fragments.

However, given the noncompliant behaviour of e-Mix178, more attention should be devoted to this matter and the tendency observed must be confirmed.

### 3.3. FTIR-ATR Spectroscopy

#### 3.3.1. PDMS

The identification and assignment of FTIR bands, by comparison with the literature, of PDMS and irradiated PDMS are summarised in Table 5.

**Figure 4:**
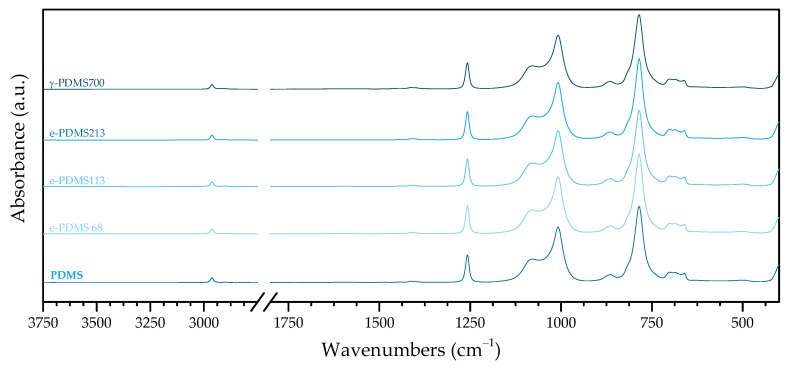
PDMS:FTIR spectra of PDMS as received, after e-beam irradiation with accumulated doses of 68, 113 and 213 kGy, and after γ-irradiation with accumulated dose of 700 kGy.

Figure 4 confirms that irradiated PDMS shows the typical fingerprint of pristine PDMS, with minimal differences between samples, being that all bands present in PDMS are also present in irradiated PDMS.

**Figure 5:**
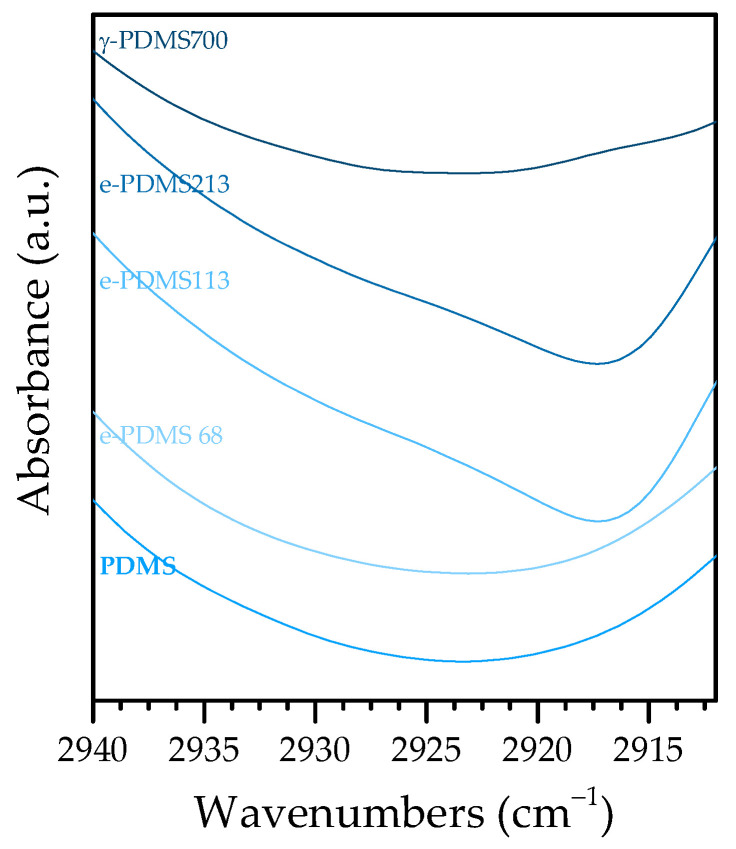
Discrete shoulder between 2927 cm^−1^ and 2917 cm^−1^ for e-PDMS113 and e-PDMS-213.

However, a possibly relevant difference is the presence of a very discrete shoulder at 2927–2917 cm^−1^ in the spectra of e-PDMS113 and e-PDMS213 Figure 5). This wavenumber is associated with an asymmetric stretching of C—H in methylene which is the result of scission of Si—C and C—H bonds and subsequent recombination to longer aliphatic chains where —CH_2_— is present, that is C_n≥2_H_n+1_, if it remains bound to the polymeric chain or C_n≥2_H_n+2_ if it forms a free alkane. This would mean that recombined aliphatic fragments would integrate the new material’s matrix or, at the very least, that free alkanes would remain trapped in it [10].

The fact that the presence of —CH2— groups occurs for e-PDMS113, e-PDMS213 and γ-PDMS700 strengthens the hypothesis of the gel point being in the previously mentioned range of 68–113kGy, meaning that a dose lower than 113 kGy would not be sufficient to induce radiolysis of methyl groups and subsequent recombination and cross-linking.

#### 3.3.2. Precursors vs. Non-Irradiated Mix

**Figure 6:**
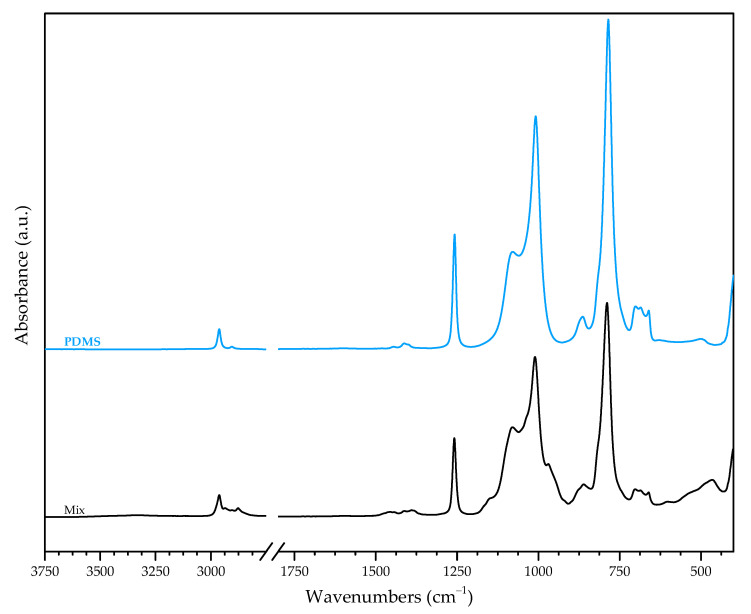
Mix:FTIR spectra of PDMS and Mix.

All typical bands present in PDMS’s spectrum are also present in the non-irradiated mixture’s spectrum as presented in Figure 6 and Table 6. Nevertheless, there are shifts in the latter, as well as when comparing to TEOS and TPOZ, namely at the following points:

- 2936, 2877, 1161, 953, 864 and 789 cm^−1^ from methyl groups;
- 1150 cm^−1^ from C—H vibrations in propoxide;
- 1010 cm^−1^, 482 cm^−1^ from vibrations of PDMS’s backbone, Si—O—Si, as well as of Si—O—Zr.

Comparison between the spectrum of Mix and the alkoxides’ shows that the most significant difference is the absence in Mix’s spectrum of some weak bands present in the alkoxides’:

- 2975 cm^−1^ (TEOS) and 2958 cm^−1^ (TPOZ), associated with methyl groups;
- 1296 cm^−1^ (TEOS) and 1297 cm^−1^ (TPOZ), associated with C—H bonds;
- 1275 cm^−1^ and 1252 cm^−1^ (TPOZ), 1100 cm^−1^ (TEOS), 1128, 1107 and 1002 cm^−1^ (TPOZ), associated to C—O bonds (first and second in propanol, third and sixth in (C—O)Zr).

At the same time, a discrete shoulder at 1033 cm^−1^ is only present in Mix’s spectrum. This is attributed to Si—O—Zr. This implies pre-irradiation reactivity of precursors, as already proposed by Gomes et al. [10] and corroborated by WDXRF results.

Bands at 466 cm^−1^ and 493 cm^−1^ are attributed to the convolution) of bands at 472 cm^−1^ from TEOS with 459 cm^−1^ from TPOZ and 500 cm^−1^ from PDMS with 482 cm^−1^ from TPOZ, respectively.

The shifts associated with methyl groups could be explained by the scission of methyl groups and recombination with other fragments or activated centres either in PDMS’s or in alkoxides’ structures. Rebonding with higher atomic mass elements would decrease the vibrations’ frequencies, since higher atomic mass shifts the vibration frequency to lower values while rebonding with lower atomic mass elements would result in the opposite effect. Another cause is the occasional substitution of Si by Zr. The most probable provenance of these Si atoms should be the silanol terminations, given the typically high reactivity of hydroxyl. Nevertheless, it could also happen in a region farther from the chain’s ending, implying that PDMS should suffer chain scission and recombination with TPOZ even before irradiation. The shifts associated with Si—O—Si bonds seem to support this possibility, since the occasional substitution of Si in Si—O—Si by Zr is compatible with the mentioned shift. The shift regarding the phonon bands follows the same type of mechanism as the substitution of Si from the PDMS chain’s backbone by Zr.

Analysing the TEOS spectrum, bands at 1073 cm^−1^ and 1100 cm^−1^ may be attributed to Si—EtOx vibrations since Si—OR groups give rise to strong bands (one or more) between 1110 cm^−1^ and 1000 cm^−1^ [44]. However, based on the study of TEOS hydrolysis [40], these bands may be attributed to Si—O—Si symmetric stretching in linear structures, indicating changes in TEOS structure before Mix preparation, as mentioned above, such as hydrolysis induced by humidity from the air [9]. Furthermore, it is evident that the absorbances associated with alkyl groups (—CH_3_ and —CH_2_—), besides shifting probably due to atomic mass effect related to the presence of Zr, tend to disappear in Mix. Likewise, the disappearing of the bands at 1275 cm^−1^ and 1252 cm^−1^ attributed to C—O stretching and shifts at 603 cm^−1^, 534 cm^−1^ and 482 cm^−1^ assigned by Colomer et al. [48] to (Zr—O)C stretching, both ensembles in propanol, indicating that this vehicle for TPOZ begins volatilising before irradiation. This event should render TPOZ more susceptible to reacting with its surroundings, causing its alteration.

In addition, the absence of most bands related to the alkoxide bonds in Mix’s spectrum is in agreement with premature modifications of the precursors, which occur previously to irradiation.

#### 3.3.3. e-Mix

Comparing Mix’s and e-Mix’s spectra, the most significant differences are the following bands only present in the spectrum of the non-irradiated mixture:

- 2936, 2877, 1471, 1456, 1365 and 1161 cm^−1^, associated to C—H in methyl and methylene groups;
- 1044 cm^−1^ and 970 cm^−1^, associated with C—O in propanol;
- 1390, 1382, 1150 cm^−1^, associated to C—H, the latter in propoxide;
- 1390 cm^−1^ and 1382 cm^−1^, associated with C—O—Si and C—O—Zr, respectively;
- 1033 cm^−1^, associated with Si—O—Zr;
- 603, 534 and 482 cm^−1^, associated with (Zr—O)C;
- 466 cm^−1^, associated with O—C—C and/or O—H.

The absence of bands related to the alkoxidic bond, as are C—O—M(M′), C—O and O—C—C, is caused by the dissociation of the ethoxides and propoxides branches in the alkoxides. This is emphasized by the loss of absorption bands attributed to C—H vibrations in methyl and methylene, meaning also dissociation of these smaller units. It is also possible that propanol volatilizes or reacts with its surroundings, explaining the lack of bands at 1044 cm^−1^ and 970 cm^−1^.

**Figure 7:**
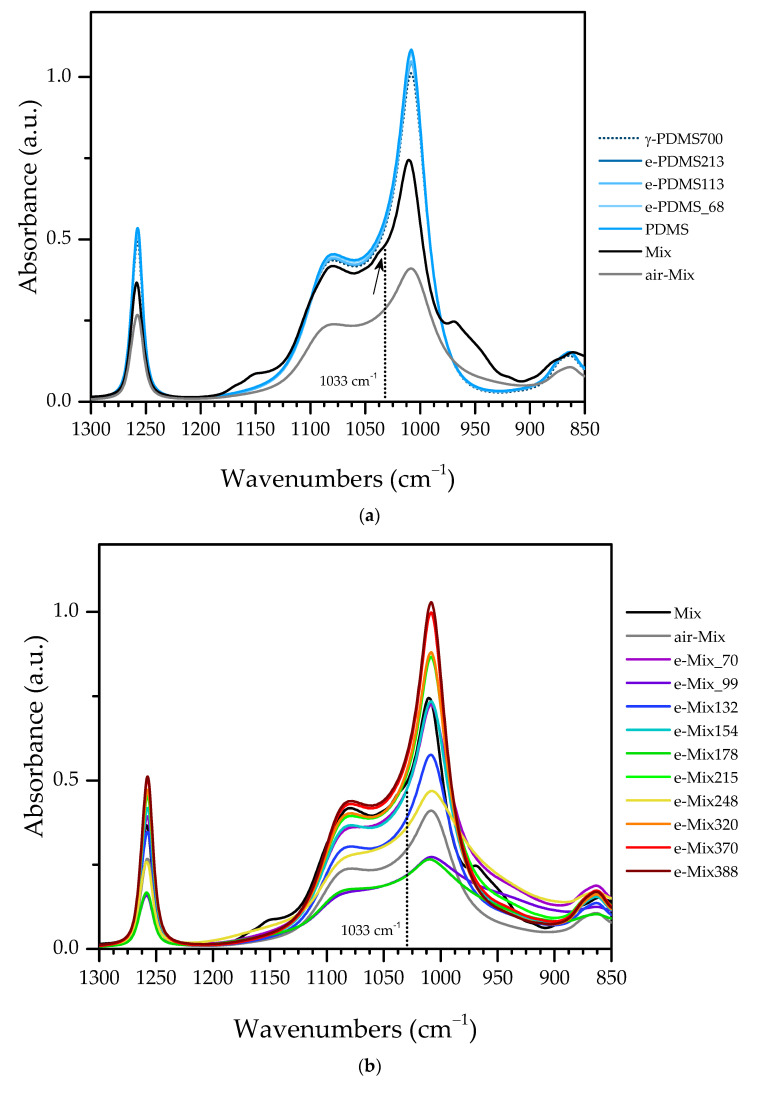

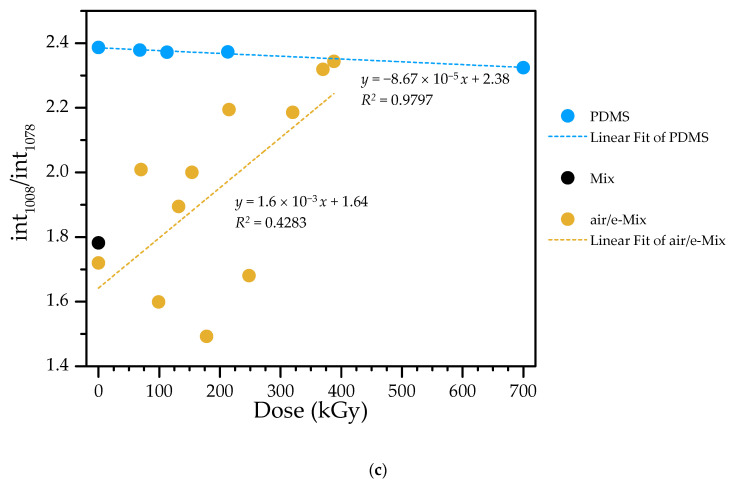
(**a**,**b**) FTIR spectra and (**c**) intensities ratio of bands at 1008 cm^−1^ and 1078 cm^−1^.

Looking with further detail at the double band at 1078/1008 cm^−1^, it is visible in Figure 7a that, besides full width at half maximum of 1008 cm^−1^ peak is higher for e-Mix, the intensities ratio between them does not remain constant, contrarily to what approximately happens with PDMS samples.

As reported by Kongwudthiti et al. [50], infrared vibrations of Si—O—Zr bonds may go from 965 cm^−1^ to as far as 1025 cm^−1^, in mixed oxides of silica-modified zirconia, depending on the concentration of Si and Zr.

In the present study, however, the ratio between concentrations of Si and Zr is much higher, as shown by WDXRF results. This, of course, should induce a shift of the band to higher frequencies due to atomic mass effect of Si. Moreover, Si—O—Zr bonds in these materials’ matrix, are part of a much larger molecule, which also influences the shift. This is in accordance with the assignment of 1033 cm^−1^ band to Si—O—Zr in Mix’s spectrum. The incorporation of higher contents of Zr in the matrix would then shift the band to lower frequencies. Considering the presence of a band at 493 cm^−1^, assigned to phonons from Si—O—Si and Si—O—Zr allied to the varying ratio of intensities of Si—O—Si asym/sym stretching double band in e-Mix, it is most likely that 1008 cm^−1^ band is the result of the sum between Si—O—Si symmetric stretching and Si—O—Zr vibrations whose frequency should fall in this same value, thus increasing its FWHM and intensity.

These data would, therefore, mean that there is the formation of Si—O—Zr bonds in the electron beam-produced matrix, attesting to the presence of a hybrid material.

Additionally, as seen in Figure 7, int_1008_/int_1078_ is very similar both for Mix and air-Mix samples and, therefore, although there is some cross-linking between PDMS and the alkoxides before processing, the lack of irradiation prevents further incorporation of Zr in the network. There is neither obvious dose effect in this ratio evolution until 248 kGy; however, after this point, the value evidently approaches the ones calculated for (γ/e-)PDMS samples. This is in agreement with gel point estimation and suggests that, beyond this threshold, the network starts being destroyed by incident radiation and degrades by scission into fragments forming few to no Si—O—Zr bonds. Furthermore, although the higher stoichiometry-favouring Zr content for remaining materials after post-irradiation processing is achieved with 132 kGy, there is also a significant increase in Zr content between 248 kGy and 320 kGy, pointing, once again, to this range of accumulated dose as optimal for inducing the formation of Si—O—Zr bonds that integrate the produced matrix. For the mentioned range, the achieved stoichiometry would then vary between 1:7.33 and 1:5.39 (Si:Zr).

**Figure 8:**
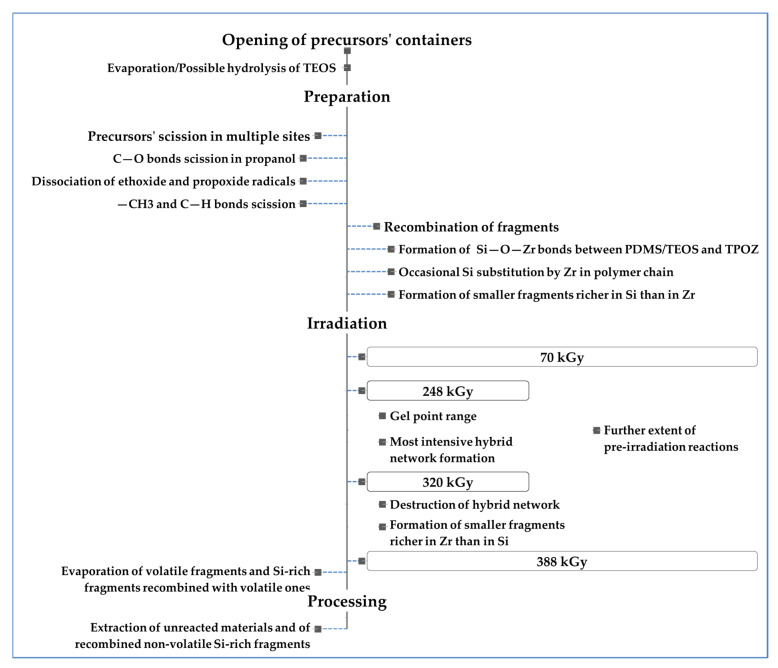
Proposed sequence of events in the production of e-HM in PDMS:TEOS:TPOZ/67:13:20 system.

Based on the presented results, the sequence of events represented in Figure 8 is proposed as a general mechanism of production of hybrid materials by electron beam irradiation in the PDMS:TEOS:TPOZ/67:13:20 system:

## 4. Conclusions

Electron beam irradiation is effective in promoting cross-linking in PDMS:TEOS:TPOZ/67:13:20 system and inducing Si—O—Zr bonds between the precursors. This results in the formation of a Class II hybrid material where covalent bonds are responsible for the network formation.

While there is some pre-irradiation reactivity between precursors, radiolysis promotes scission on multiple sites of precursors’ structures, which induces hybrid network formation to a greater extent than that if sol-gel method was used, since the latter would promote cross-linking mainly originating on silanol terminations of PDMS. This system’s gel point is estimated to be achieved for the dose range of 248–320 kGy.

Even though in-air curing is attainable, the degree of formation of Si—O—Zr bonds is heavily reduced and, as such, Class II hybridisation between the polymer and the alkoxides cannot be proven for these conditions.

While there is an evident effect of the irradiation in the structural hybridisation mechanism, there is not an obvious dose effect on the extent of these reactions, i.e., there is not a linear—neither direct nor inverse—correlation between absorbed dose values and direction or magnitude of reactions. Stopping time between partial irradiations may prevent the linear evolution of the studied parameters. Further studies should be carried out to clarify this point, as well as to address the reproducibility of results correlated to the studied doses. It is also important to introduce the temperature variable and understand its role in the process, as well as to gather more information about the material’s morphology through SEM studies. Moreover, after establishing a production procedure, extensive characterisation and research should also be carried out regarding the produced material’s application, namely as a multifunctional conservation-restoration product.

## Figures and Tables

**Table 1:** Description of samples’ name coding.

Field	Information	Code	Description
Prefix	Curing method	[none]	Not cured
		air	In-air curing
		e	Irradiation with electron beam
		γ	Irradiation with gamma photons
Root	Composition	PDMS	Polydimethilsiloxane as received
		Mix	Non-irradiated PDMS:TEOS:TPOZ with 67:13:20 m% concentration, as of preparation
Suffix	Accumulated dose (kGy)	[numeral representing dose]	Accumulated dose in kGy

**Table 2:** Calculated gel fraction in mass percentage (m%).

Material	Dose (kGy)	Gel Fraction (m%)
e-PDMS	68	89.5
	113	99.5
	213	99.4
air-Mix	0	57.3
e-Mix	70	56.4
	99	65.3
	132	55.3
	154	57.5
	178	62.7
	215	60.8
	248	53.8
	320	85.5
	370	71.5
	388	83.8

**Table 3:** Si and Zr content (Despite IUPAC recommendations ([35], p 97), WDXRF results are presented in percentage of weight (wt%) as this is the unit indicated by the equipment’s software.) in (air/e-)Mix samples, normalised to 100% and Si:Zr mass and atomic fraction (χ_m_ and χ_at_).

Dose (kGy)	Si (wt%)	Zr (wt%)	Si:Zr (χ_m_)	Si:Zr (χ_at_)
Mix	* 82.87	* 17.13	4.84	15.71
air-Mix	74.0	26.0	2.85	9.24
e-Mix70	57.2	41.9	1.37	4.43
e-Mix99	79.7	19.7	4.05	13.14
e-Mix132	69.1	30.2	2.29	7.43
e-Mix154	65.0	34.3	1.90	6.16
e-Mix178	56.9	42.1	1.35	4.39
e-Mix215	83.3	16.1	5.17	16.80
e-Mix248	79.6	19.8	4.02	13.06
e-Mix320	55.4	43.7	1.27	4.12
e-Mix370	63.4	35.9	1.77	5.74
e-Mix388	77.5	22.5	3.44	11.19

* Theoretical values calculated for a mixture of PDMS:TEOS:TPOZ with 67:13:20 m% proportions and normalized to 100%.

**Table 4:** Si and Zr content in (air/e-)Mix samples after post-curing processing, normalised to 100% and Si:Zr mass and atomic fraction (χ_m_ and χ_at_).

Dose (kGy)	Si (wt%)	Zr (wt%)	Si:Zr (χ_m_)	Si:Zr (χ_at_)
Mix	* 82.87	* 17.13	4.84	15.71
air-Mixp	55.4	43.7	1.27	4.12
e-Mix70p	57.2	47.3	1.21	3.93
e-Mix99p	68.5	52.7	1.30	4.22
e-Mix132p	46.4	52.7	0.88	2.86
e-Mix154p	52.3	46.8	1.12	3.63
e-Mix178p	65.5	33.9	1.93	6.28
e-Mix215p	55.2	43.7	1.26	4.10
e-Mix248p	68.8	30.5	2.26	7.33
e-Mix320p	61.9	37.3	1.66	5.39
e-Mix370p	64.8	34.5	1.88	6.10
e-Mix388p	88.3	11.7	7.55	24.51

* Theoretical values calculated for a mixture of PDMS:TEOS:TPOZ with 67:13:20 m% proportions and normalized to 100%.

**Table 5:** Assignment of FTIR bands for PDMS before and after irradiation.

Frequency (cm^−1^)			Assignment	References
PDMS	e-PDMS	γ-PDMS		
2962/2905	2962/2905	2962/2906	C—H asym/sym stretch in Si—CH_3_	[36,37,38]
Background overlap	2927–2917 (113 kGy, 213 kGy) Background overlap (68 kGy)	2933	C—H asym stretch in —CH_2_—	[39,40]
1600	1600	1607	adsorb H_2_O bend	[41,42]
1480/1373	1481 (68 kGy) 1484 (113 kGy, 213 kGy)	1480	C—H asym/sym bend in —CH_3_ and/or —CH_2_—	[39]
1445	1445 (68 kGy) 1446 (113 kGy, 213 kGy)	1445	C—H asym bend in —CH_3_	[39,40]
1412	1412	1412	—CH_3_ asym bend in Si—CH_3_	[43]
1402/1258	1402/1258	1402/1258	—CH_3_ asym/sym bend in Si—CH_3_	[41,42]
1258	1258	1258	—CH_3_ sym bend in Si—CH_3_	[38,43]
1078/1008	1080/1008	1078–1080/1008	Si—O—Si asym stretch in linear structures	[40,44,45]
864	864	864	Si—CH_3_ rock in PDMS	[43]
785	785	785	SiO_4_ asym vibration, Si—O—Si bend in SiO_2_, Si—CH_3_ rock, CH_3_ rock	[40,44,46]
500	500	502	Si—O—Si bend, phonons from M—M and M—O—M′ (M=Si, M′=Zr)	[47]

**Table 6:** Assignment of FTIR bands for precursors, Mix and e-Mix.

Frequency (cm^−1^)					Assignment	References
PDMS	TEOS	TPOZ	Mix	e-Mix		
		3344	3345	3500	O—H stretch in H-bonded OH/propOH	[39,40]
2962/2905			2962/2905	2962/2905	C—H asym/sym stretch in Si—CH_3_	[36,37,38]
	2975	2958	-	-	C—H asym stretch in —CH_3_	[39,40,48]
	2929	2933	2936	-	C—H asym stretch in —CH_2_—	[39,40]
	2889	2873	2877	-	C—H sym stretch in —CH_2_—/—CH_3_	[39,40,48]
1600			1603	1600–1634	adsorb H_2_O bend	[41,42]
1480	1484	1470	1471	-	C—H asym bend in —CH_3_ and —CH_2_—	[39]
		1458	1456	-	C—H asym bend in —CH_2_—/—CH_3_ (in propOx from TPOZ)	[39,48]
1445	1444	1438	1443	1445	C—H asym bend in —CH_3_	[39,40]
1412			1412	1412	—CH_3_ asym bend in Si—CH_3_	[43]
1402/1258			^1^/1258	1404	—CH_3_ asym/sym bend in Si—CH_3_	[41,42]
1373(?) ^2^	1391	1380	1390 1382	-	C—H sym bend in —CH_3_ and/or in propOx and propOH	[39,40]
	1391	1380	1390 1382		C—O—M stretch, with M=Si in TEOS and M=Zr in TPOZ	[49]
	1366	1364	1365	-	C—H wag in —CH_2_—	[40]
	1296	1297	-		C—H wag/twist	[40]
		1275/1252	-		C—O asym/sym stretch in propOH	[48]
1258			1258	1258	—CH_3_ sym bend in Si—CH_3_	[38,43]
	1168 1100/1073 959		1161 1096/1080 953	- -/1078 -	Si—OCH_2_CH_3_	[44]
	1168		1161	-	C—H rock in —CH_3_	[40]
		1154	1150	-	C—H wag/twist in propOx	[48]
	1100/1073		1096/1080	-/1078	Si—O—Si asym/sym stretch in linear structures	[40,44,45]
	1100	1107	-	-	C—O asym stretch	[40]
		1128	-	-	(C—O)Zr asym stretch and «skeletal stretches» combination	[48]
1080/1008			1080/1010	1078/ 1010–1008	Si—O—Si asym stretch in linear structures	[40,44,45]
		1045 1012 968	1044 1010 970	- 1008 -	C—O stretch in propOH	[48]
			1033	-	Si—O—Zr	[50]
		1002	-	-	(C—O)Zr sym stretch	[48]
		968	970	-	C—O stretch in propOH	[48]
	959		953	-	C—H rock in —CH_3_, Si—O(H) stretch	[40,48]
864		862	861	864	Si—CH_3_ rock in PDMS, C—H twist in propOx	[43,48]
	811		813	813	—CH_2_— rock	[40]
785	785	782	789	789–785	SiO_4_ asym vibration, Si—O—Si bend in SiO_2_, Si—CH_3_ rock, CH_3_ rock	[40,44,46]
		596 545 498	603 534 482	- - -	(Zr—O)C stretch	[48]
500			482	493	Si—O—Si bend, phonons from M—O—M and M—O—M′ (M=Si, M′=Zr)	[47]
	472		466	-	O—C—C bend	[40]
		459	466	-	O—H wag/twist	[51]

^1^ Band masked by band present at 1390 cm^−1^. ^2^ Very weak shoulder.

## Data Availability

The data presented in this study are available on request from the corresponding author.

## Appendix A. Preparation of the Mixture to Be Irradiated

In this type of operations, there are several parameters that can affect the final result, which was intended to be a homogeneous mixture without distinction of phases, with the desired proportions between the selected precursors. In this section, the results for the various parameters tested are presented.

Since it is a ternary mixture, the parameter that is most easily identified is the order in which each reagent is added. Others, although less obvious, are equally essential, namely the type of material and method used in transferring the reagents.

### Appendix A.1. Transfer Material and Method

Due to PDMS’s high viscosity and a non-Newtonian behaviour of TPOZ, transfers of these precursors were made by decanting or pouring, controlling the necessary quantities through the measurement of the poured mass. For mixing containers, polyethylene (PE) tubes with lids were also selected, so that they could be easily shaken using the vortex mixer without having to resort to placing magnets inside. The diameter of the tubes is 3 cm and easily allows both the inlet and the flow of reagents and mixtures.

### Appendix A.2. Reagent Addition Sequence

Metal alkoxides, with the exception of silicon alkoxides, are highly reactive. For this reason, when in contact with PDMS, TPOZ can cause hydrolysis and consequent precipitation of oxy-polymer prior to irradiation [9,52].

It was therefore considered that the contact between these two precursors should be postponed as long as possible and that, at that time, care should be taken to ensure that the dispersion of the third component in the mixture of the first two takes place as quickly as possible in order to avoid the formation of heterogeneous agglomerates as a result of hydrolysis followed by precipitation. With this premise in mind, there were two possible combinations for mixing the reagents: (A) first mixing TEOS and TPOZ_PropOH_, adding PDMS last; or (B) mixing the PDMS and TEOS first, followed by adding the TPOZ_PropOH_ to that mixture.

#### Appendix A.2.1. Method A

An amount of 2.5 mL of TEOS was mixed with 5.0 mL of TPOZ and PDMS was added, pouring it from a beaker, on the scale, in aliquots of ~3 mL each, until the mass of 12.2(6) g was reached, which corresponds to the selected concentration of 67:13:20 m% for PDMS:TEOS:TPOZ, respectively. The addition of PDMS was alternated with stirring. This was carried out by alternating mechanical agitation with a vortex mixer with agitation by ultrasounds with the vessel in a water bath, for five cycles of 3 min each. In this way, the mixture was obtained more easily, but still with the formation of two distinct phases that were manifested by small turbid regions within the TEOS:TPOZ mixture. For this reason and seeing that the application of ultrasounds favoured homogenisation, the application of ultrasounds was resumed for ~80 min, continuously. The mixture was then clear, transparent and homogeneous, with a yellowish tone.

#### Appendix A.2.2. Method B

An amount of 3.9 g of TEOS, ~5 mL at a time, was added to 20.1 g of PDMS, alternating with vortex agitation. The solution was practically clear, with only a few bubbles, apparently of air, very small and homogeneously dispersed, which disappeared after few minutes. Afterwards, 8.57 mL of a 70 m% solution of TPOZ in ethanol were added, until the mixture reached 32.57 g, which corresponds to the selected concentration of 67:13:20 by mass for PDMS:TEOS:TPOZ, respectively. A TPOZ bubble was formed with practically its entire volume being immersed in the PDMS:TEOS mixture. It was left for ~80 min in the ultrasonic bath. After this time, the reagents were not mixed. The vial was inverted and the gelled interface was broken, revealing a large lump in the centre. With agitation and ultrasounds, it was homogenized. This operation took ~40 min longer. In the end, a transparent solution with a yellowish tone was obtained, but with some regions slightly more turbid, from which it can be deduced that the homogenisation was not complete. Apparently, these corresponded to the interface between the formed TPOZ bubble and the PDMS:TEOS mixture. When touched, the temperature of the mixture container was higher, however, it was not possible to measure it at the moment.

It is concluded, therefore, that the best way to obtain a homogeneous mixture, without separation of phases that are visible to the naked eye or occurrence of early precipitation of oxy-polymer, is method A; that is, first mix the TEOS and TPOZ_PropOH_ and then add the PDMS to this mixture.

## Appendix B

### Sample Positioning and Dosimetry

Samples were positioned on top of a PS support at Z = 16 cm from beam exit, corresponding to the minimum distance between the sample and the beam exit with maximum homogeneity of dose distribution to the entire volume.

Simulation with ESTAR: stopping power and range tables for electrons from the National Institute of Standards and Technology [32] showed that, assuming 0.001205 g·cm^−3^ as the density of dry air at 20–25 °C, and 0.99238 g·cm^−3^ as the PDMS:TEOS:TPOZ mixture’s density, beam range is ~50 m and 5.1 cm in each material, respectively, confirming the beam can reach the sample and cross it with neglectable energy loss.
